# The Great Extension at Leeds

**Published:** 1916-10-21

**Authors:** 


### THE GREAT EXTENSION AT LEEDS.

An important part of the great extension to Leeds General Infirmary, which was undertaken as a memorial to the late King Edward VII., is virtually completed, and forms a landmark in the history of that widespread hospital reconstruction which, we may be permitted to remark, has accompanied the three series of Reports on Hospitals of the United Kingdom which have from time to time appeared in our columns. On the particularities of the new hospital block, which will immediately provide for a hundred wounded soldiers, we need not now dwell, since this block should be considered in conjunction with the ambitious scheme for the fine new operating theatres, of which the completed wards are only the first part. But it may be frankly admitted that the theories of planning, construction, and ventilation embodied in the new buildings are full of matter which is suggestive in innumerable ways to all who have to make a study of hospital architecture and construction. These arts and sciences, for they partake of the nature of both, minister to efficient administration, the object of which, it is well to remind ourselves in the midst of details, cost, and specifications, is first and last the well-being of the individual patient.

The larger part of the whole matter is that Leeds General Infirmary presented a problem now familiar—namely, the general question as to whether or no reconstruction should be interpreted to mean complete rebuilding or the enlargement and adaptation of the old. Such a question is always complex. Site and finance, as well as architectural problems, are involved, and the difficulties surrounding such transformations are so great that managers who have to face them can almost be excused a fear of embarking on the complications which surround them at every turn. The successful handling of intricate problems such as these demands not merely a high order of business capacity, probity, and acuteness of intellect on the part of the members of the hospital board of management; it also requires of them long periods of hard work, snatched often from their hours of leisure after a day's work at their own businesses. It is one of the characteristics of British public life in which the genius of our people is best illustrated that men can be found to fulfil these conditions and to bring to fruition great schemes such as that now evolved at Leeds.

Under a voluntary hospital system like the one of which Englishmen are justly proud it is always difficult to maintain a hospital standard, since the necessary driving power is decentralised and dependent upon the energy and resource of the men who run the different institutions. With the freedom of initiative thus created a competition in well-doing is essential to progress; but there is apt to be pre-occupation with local needs (and difficulties) for lack of a recognised central body charged with the maintenance of a standard throughout the hospitals as a whole. In London, of course, King Edward's Hospital Fund largely supplies this centre and can enforce a standard by the power of the purse. In the provinces, where there is no King's Fund, matters are more complicated, and the difficulty is to provide some means to fill this gap. It was with a sense of the need, and of the responsibility for supplying it, that The Hospital's series of Reports was conceived and undertaken; and it is in the light of the claims there made that these subsequent extensions and reconstruction schemes can be most illuminatively studied. Such a study proves that the feeble cry (which greeted their commencement) that cash not criticism was what the hospitals required was as short-sighted as it was short-lived. It proves, too, that, judged by the test of experience, criticism at once clear and constructive is the necessary prelude to the awakened conscience, which alone can provide the energy and funds required for such a great scheme as this which the Leeds General Infirmary and its managers are now bringing towards its ambitious and early conclusion. They have their reward, and may hope, we are confident, for the success of the final appeal for £30,000 which will shortly have to be made.

